# The Future of the Infirmary

**Published:** 1921-07-09

**Authors:** 


### The Future of the Infirmary.

AT the West Midland Poor-law Conference, held at Malvern, Mr. William Darby, late Chairman of the Birmingham Board of Guardians, read an interesting paper. Though it was called "Poor-law Reconstruction in Relation to Health," it really dealt with the development now required of the Poor-law infirmary, and the relation of the infirmary to the voluntary hospitals. He began by saying that voluntary hospitals serve large areas beyond the towns where they are situated, and even before the war were financed with difficulty. It is doubtful if all will be able to continue. Their present demand for maintenance charges from patients coming from districts outside the Hospital Saturday collection makes it more difficult for the poorer patients to get special medical attention. This leads him to expect an increased demand on the services of the Poor-law infirmary. It seems clear that a larger development is essential. One of the early duties of the Poor-law was to provide for confinements, many of them for illegitimate births. But since the war the Guardians have been asked to provide extensive accommodation for married women. He believes that this demand will survive the present shortage of houses, and that the larger infirmaries will have to possess women's departments. Greater care of the unmarried mother is necessary, and greater care and after-care of babies. Even in the best institutions the mortality rate is very high; it is made, indeed, a common charge against the Guardians. To meet the new demands for treatment of chronic, phthisical, venereal, and mentally defective cases the Unions must either amalgamate, co-operate for special work, or make contracts between themselves for its accomplishment. The Guardians must share in the work of reconstruction.

